# The perspective of staff members of two forensic psychiatric clinics in German-speaking Switzerland on the introduction of recovery orientation: An explorative study

**DOI:** 10.3389/fpsyt.2022.946418

**Published:** 2023-01-09

**Authors:** Susanne Schoppmann, Joachim Balensiefen, André Nienaber, Stefan Rogge, Henning Hachtel

**Affiliations:** ^1^Department of Education, Research and Practice Development, Universitäre Psychiatrische Kliniken (UPK) Basel, Basel, Switzerland; ^2^Nursing Directorate, Universitäre Psychiatrische Kliniken (UPK) Basel, Basel, Switzerland; ^3^Zentralinstitut für Seelische Gesundheit (ZI) Mannheim, Mannheim, Germany; ^4^Forensic Department, Universitäre Psychiatrische Kliniken (UPK) Basel, Basel, Switzerland; ^5^Forensic Department, Faculty of Medicine, Universitäre Psychiatrische Kliniken (UPK) Basel, University of Basel, Basel, Switzerland

**Keywords:** personal recovery, forensic inpatient psychiatry, focus groups, staff attitudes, obstacles and challenges

## Abstract

Recovery orientation (RO) is directed at living a worthwhile life despite being impaired by the constraints of mental illness. Although being quite common in general psychiatry in Switzerland, the dual mission of forensic psychiatry—safeguarding and therapy—challenges the idea of establishing RO as a work philosophy in this context. This explorative study qualitatively investigates baseline expectations and professional perspectives of forensic staff members concerning the idea of establishing RO in Swiss forensic psychiatric wards. Thereby, three central themes were worked out, namely “challenges associated with recovery,” “expected barriers,” and “possible recovery-oriented interventions.” From a general point of view, the staff members were uncertain whether RO interventions could be introduced at all, and if so, to what extent. This, on the one hand, had to do with structural obstacles such as security requirements; however, personal obstacles in the form of different, sometimes contradictory attitudesand ideals and fearful anticipations—such as the loss of authority and power—also played a central role. As forensic psychiatric wards are non-existent in Latin-speaking Switzerland, the study does only refer to the German-speaking language region.

## Introduction

Personal recovery can be seen as an individual journey (1) that enables persons with mental health disorders to live a meaningful and self-determined life. Davidson et al. (2), for instance, defined recovery to be “what a person with a mental illness does to manage his or her condition and reclaim his or her life from the distress and wreckage the illness, and the stigma and discrimination associated with having the illness, may have caused.” Thereby, the central elements of recovery reflect basic human needs, such as “being connected with others,” “having hope and optimism for the future,” “gaining (or rebuilding) a positive sense of identity,” “being empowered to make own decisions,” and “discovering life as meaningful.” These needs are represented in the CHIME model, which conceptually includes the corresponding elements “connectedness,” “hope,” “identity,” “empowerment,” and “meaning” (1). As a philosophy of work, recovery orientation (RO) in terms of employment of peer workers is widely established in general psychiatry in German-speaking Switzerland (3, 4). Nevertheless, in contrast to other countries, secure recovery (5)—that is, using recovery principles in forensic mental health—is presently not systematically introduced (6–11). As a previous study has shown, a central problem regarding the introduction of RO in secure wards is the difficulty for forensic inpatients to develop a confidential relation with the staff due to the extensive imbalance of power (12). However, at the same time, staff members often form the patients’ only social contact and thus play a very important role in facilitating recovery (8, 10, 11). The staff, on the other hand, has to deal with the dual mission of forensic psychiatry, namely the contrast between therapy and safeguarding society from possible threats through the criminal behavior of these patients (13). This tension may cause inner loyalty conflicts that result in cycling between control and care (14). Correspondingly, in the context of a concept analysis of restrictiveness, Tomlin et al. (15) showed that being either caring or patronizing is an inherent aim of the forensic system; thereby, for instance, patients described staff members as *key-holders, lacking in empathy, insensitive, disempowering, forceful, abusive, prone to over-reaction* [(16), p. 34] with regard to patronization. To some extent, these attributes were reflected and confirmed as marks of patronization in focus groups with forensic inpatients in German-speaking Switzerland (12). In order to promote RO in forensic psychiatry despite these constraints, Drennan and Wooldridge (9) identified five central dimensions, which are essential to this end, namely the support of recovery along the care pathway, the enhancement of quality of relationships, the management of risk and security, the creation of opportunities for a “life beyond illnesses”-perspective, and peer support. These dimensions reflect central starting points on the abovementioned way to recovery in forensic psychiatry and raise the question of how to establish them as means for orientation in Swiss forensic wards.

This article systematically reports the contents of eight focus groups that were held on six wards in two forensic psychiatric university hospitals in Switzerland (Psychiatrische Universitätsklinik Zürich (PUK) and Universitäre Psychiatrische Kliniken (UPK) Basel). These hospitals treat mainly patients suffering from psychotic disorders on medium secure units. The focus groups were conducted between February and June 2020 and are part of a larger ongoing project under the label of RE-FOR-MA (Recovery Orientation in Forensics and Secure Settings), which is funded by the Swiss Federal Office of Justice. In this study, the reported results of the focus groups represent the starting point of the larger project RE-FOR-MA.

In the context of this project, the focus groups are part of a mixed-methods approach (17), which has its root in pragmatism (18–20) and combines qualitative and quantitative data in order to gain a more profound understanding of the research topic. The aim of this project is to raise knowledge regarding the applicability of RO attitudes and interventions in forensic wards and the effectiveness of these adjustments on several factors of therapeutic relevance in forensic mental health institutions in Switzerland. It uses patient and staff collaboration in the development and implementation of recovery-oriented interventions specific to each ward. To this end, focus-group interviews with patients and staff, as well as different surveys, will be conducted again after the completion of the implementation phase. The Ethics Committee Northwest and Central Switzerland confirmed the fulfillment of the general ethical and scientific standards for research with humans.

## Materials and methods

The focus groups were performed following an RO-training session in each of the participating wards, during which two research fellows (SSc and JB) had already established contact with some of the focus-group participants. In these 2-h sessions, RO was introduced to patients and staff members together, based on the theoretical principles of the CHIME framework (1) and a vivid case report. The principles of the CHIME framework were explained and illustrated with a biographic narrative from a former forensic patient who is working as a peer in forensic psychiatry in the Netherlands. The sessions were also used to recruit participants for the focus groups. All participants received oral and written information about the study and were included if written informed consent was provided. The focus groups took place during regular working hours of the staff.

### Theoretical background

As a theoretical background for executing the focus groups, the documentary method (21, 22)—which is closely linked to reconstructive social research—was used. Thereby, it is assumed that the group members implicitly have a commonly shared knowledge specific to a certain milieu. Against the background of the social interview situation and a general topic, this shared knowledge is being unfolded in a self-directed, discursive manner, which should not be disturbed by an interviewer (22). Thus, the interviewer only presented the topic at the beginning and sporadically intervened with questions of clarification and short summaries if necessary. In addition to the identification of the conjunctively shared knowledge as the targeted subject of interest, the documentary method also allows to differentiate between the narratives of single interviewees. Originally, the documentary method aimed at deducing theories from the material through an inductive analytical process that results in generalized patterns or types (21). However, thematic analysis was used (23, 24) for the analyses of the focus-group protocols—which is also applicable to data generated in focus groups (25)—because our research focuses on exploring perspectives of forensic mental health staff related to RO, and not on deducing a specific theory.

### Participants

We conducted eight focus groups with staff members, which were employed either at the Universitäre Psychiatrische Kliniken (UPK) Basel or the Psychiatrische Universitätsklinik Zürich (PUK); each of these hospitals was represented by three medium secure forensic wards, whereas one of the wards in Basel was for adolescent patients. The offer to participate in a focus-group interview was directed at all employees of the six wards, for which four appointments were made available in each case. A total of 50 employees, about half of the employees in these wards, took part in the focus-group interviews. This corresponds to an accidental or convenience sample since the employees’ motives for or against participating in a focus-group interview were not made explicit. Table 1 gives an overview of the participants and their occupations and shows that the occupational groups are distributed approximately equally in proportion to their occurrence by chance. Between five and nine staff members participated in each focus group. This size corresponds to the recommendations of Krueger and Casey (26). Since the interest to participate among staff members in two wards in Basel was too high to comply with this recommended size, two focus groups were performed in each ward. In total, 26 female and 24 male staff members were present in the focus groups. The age of the participants in both clinics is between 19 and 63 years. The median of the years with professional experience is 4.25 in the one and 4.5 in the other clinic. Table 1 gives an overview of the participants and their occupations.

**TABLE 1:** Overview of participants in dependence on their occupation.

Professional group	Participants
Medical service	4
Nursing service	34
Psychological service	7
Social service	2
Occupational therapists	3

### Procedures

#### Proceeding in the focus groups

The interviews were held on premises that belong to the hospitals. Two research fellows—one as an interviewer (SSc) and one as an assistant and observer (JB) (26)—led the focus groups. Apart from some of the participants in Basel, who knew the interviewer casually from working in the same institution, most of them had seen the research fellows for the first time in the preceding training session. Before the focus groups started, the participants gave their written informed consent. The focus groups lasted between 60 and 75 min and were audiotape-recorded. At the beginning, the participants were informed that the purpose of the interview was rather to get into a thematic discussion with each other than to answer specific questions. All focus groups were initiated with the following statement: “You all participated in the training session, which introduced the concept of Recovery Orientation (RO). When you think about it, what goes through your mind?” During the focus groups, the participants quickly came into vibrant discussions, so that little interventions by the interviewer were necessary.

At the end of the focus groups, the participants were informed that they would receive the results of the analyses in a few weeks. The research fellows (SSc and JB) made field notes and sketches of the seating arrangements immediately afterward.

### Data analysis

All focus groups were transcribed verbatim. The analyses of the focus groups were performed by applying thematic analysis (23) in an inductive way. The transcripts were collated with the audio files to control for transcription errors. All passages were coded, and the codes were collated to find themes and subthemes for the particular transcripts. Finally, the whole data set was examined for thematic similarities and differences; thereby, themes were repeatedly adapted or changed in discussion with the research team (SSc, JB, and HH).

### Trustworthiness

Two of the researchers (SSc and JB) analyzed all of the transcripts independently. At all stages of this process, reflecting writing (27) was used in order to become aware of the researcher’s own experiences. As the results were presented to the staff members’ a few weeks after the focus groups, they confirmed their accordance with their own impressions. In preparation for the article, we applied the COREQ criteria (28). The authors of this article have translated all of the following quotations in an attempt to reflect the participants’ original wording.

## Results

In all focus groups, the participants strived for clarification regarding the meaning of RO in a forensic setting. They began to discuss the challenges they associated with RO, the hindrances they expected, and the RO interventions that were possibly about to be implemented in the wards. These global themes included several subthemes; an overview is given in Table 2.

**TABLE 2:** Overview of themes and subthemes.

Challenges associated with RO	Expected obstacles	Possible recovery oriented interventions
Tension between security & therapy	Stigma	Resource-orientation
Social connectedness	Structural obstacles	Participation
Hope	Personal obstacles	Ward rules
Identity and finding meaning		Success-stories & stimulation
Empowerment & participation		Creating a therapeutic milieu
Trust		Shaping transitions

### Challenges associated with RO

#### Balancing the tension between security and therapy

The tension between the therapeutic obligation toward the patients and the role of a safeguarder of society was a subject of debate in all focus groups. Thereby, staff members tended to consider the societal demands regarding a successful recovery of psychiatric forensic patients as delusive. In their view, the corresponding criteria may unnecessarily prolong the stay in inpatient forensic treatment:

*“Yes, so what is demanded from such a person, eventually that is important, isn’t it? What does society, the society provide for a framework, for such a patient, well, it is. and the constraints we make. or does treatment take a little longer then? Is it possible at all, is it feasible, and is it tenable, that is also important* (…*). The question simply is, what standards are expected from such a person, what must he fulfill to satisfy all the requirements, and then to be able to integrate in society* (…*)”* (FG 1, L 41 - 49).

The responsible authorities and subordinate institutions were primarily understood as representatives of the civic interests of security, which clearly emphasized the perception of having the mission to secure the mandate to help. From a therapeutic perspective, the participants perceived this focus as unsatisfying, preventing potentially beneficial interventions in terms of positive risk-taking. Nevertheless, the participants also highlighted the necessity to find a realistic balance between the orders of securing and treating and the possibility of understanding security and therapeutically reasonable interventions as complementary.

In that light, it is important to keep in mind that ignoring the legal system’s framework in the therapeutic relationship might be harmfully disappointing to the patients:

*“I recognize a new perspective* (…) *with recovery now, I think that’s great. I think you have to be very sensitive and careful how you approach it. If it becomes a farce or a mockery, and the forensic aspect is dominant again in the end; or you forget about that, because you have a good working relationship with the patient, and then, in the following institution, you’ll have to say: ‘Oh no, there is the offense, after all’, and then the external person says:, ‘sorry, even if it went all well, but the offense!’ And then the perspective is limited again, I think that would be like extra hard for the patient”* (FG 4, L185 -192).

#### Social connectedness

Stable and emotionally meaningful relations with other people are one of the most central ingredients of recovery, as they form the backbone of a fulfilling life beyond illness; the implementation of such relations was considered to be a challenge:

*Speaker 1: Or especially with connectedness, I have the impression that it often is the case with schizophrenics that they don’t have any relationships. More often than other populations. And, or there is not the desire for a relation. And it is difficult, then. Yes, basically it is something of importance for a human being, but*… *for instance, I have patients who have zero interest to interact with someone else.*

*Speaker 2: Yes, it becomes difficult, too, just partly come back into the old conditions, where the offences often took place in the relations. That is just*… *one might freeze if one knows, when he says, preferably he would like to get back where before, yes, very bad stories went down. One has to say then: “That probably is not quite perfect: Yes, if you think so [referring to the anticipated answer of the patient; laughing]” Yes, like this*…*” (FG 6, Z. 308 – 319)*

In the focus groups, many difficulties regarding the social participation of patients were discussed, such as the fact that some of the patients would show no desire for connectedness and interaction at all. Others, on the other hand, would be unable to accept the perniciousness of their old social environment and refuse to see the impracticability of continuing to live the “old social life” as if nothing had happened. Symptoms of psychiatric disorders and an unfavorable social environment are classified as dynamic risk factors in forensic psychiatry. As the quotation above shows, both the reluctance and the motivation to engage in social relations challenge the staff because it might be necessary to motivate the patients in one case and to slow them down by inviting them to critically examine their unfavorable environment in the other.

Moreover, the participants described motivational work as being particularly important:

*“But otherwise, in principle, that one motivates individual patients, clients, that one motivates individuals to resume contact, and they then receive feedback, because suddenly a letter comes, or a telephone, or oh là là, after a year a visit, who is suddenly at the door, these are good things, if it suits both sides. That is important. And then you have to search, who might be suitable”* (FG 6, L 506-510).

Finally, the staff members saw room for improvement not only concerning social relations between patients and people from outside the wards but also between the patients in the wards and between the patients and the staff members, for example, throughout joint leisure activities.

#### Hope

With respect to “hope,” two dimensions, which should thematically be distinguished, were present in the focus groups. For one thing, the staff members associated hope with the introduction of RO concerning an improved or modified operating modus of the wards. Moreover, they also associated hope with a possibly brighter future for their patients throughout RO.

Concerning the latter, the participants suggested that introducing RO will make interdisciplinary cooperation more vibrant and will enable them to recognize the fruits of their own work in a more striking way:

*“I also think it’s nice to be able to see, afterwards, that you’ve achieved something together. In our profession, you go home at the end of the day - a carpenter sees ‘I’ve made a table today’. And in our case, that’s not so much like that. It takes a little more time and it would be nice to see what you have achieved, together with the patients.* (…) *we as a team do not tell them what to do from above, but that it is together”* (FG 7, L. 204 – 209).

Furthermore, the staff members suggested that a more dense commonality with the patients would increase cooperation between patients and staff, which then again might serve as a bedrock for assigning more responsibility to the patients; this responsibility is related to compliance with ward rules in particular.

*“Rules that apply on the ward. We have already worked them out as much as possible together with the patients. And now, for us, it is actually, how should I say, I hope that now, for example, a change is suggested to these house rules and that the patients themselves take responsibility to enforce this. At the moment, it is often our responsibility that we comply to the rules or that they comply to the rules. And that’s what I’m actually hoping for from Recovery, that we’ll get to the point where we can say, hey, these are our rules that we’ve set up and we’ll also make sure that they’re maintained”* (FG 7, L 153 – 161).

Finally, few staff members expressed some optimism that the clinic management would help them introduce RO, whereas its role was seen in a rather ambivalent manner in general. Especially concerning the idea of providing more possibilities for participation, the management was described as being overcautious in a therapeutically counterproductive way.

Regarding the hope for the patients’ future, there was a clear desire for change among the staff members directed at the general situation in the forensic setting, which was in itself considered to be a source of hopelessness for the patients:

“*And I think that the longer people are in a setting like this, the more resigned they become, and there is a hopelessness, a lack of perspective. Many things that patients normally take for granted are taken away from them, additionally. And that is always such an act of balancing. And that is also incredibly demanding, yes. And they have to have something where they see the purpose again, some kind of meaning. And they also realize, ‘I somehow benefit from that”’* (FG 8, L 89-95).

For the evocation of hope, it would be central that the patients can make the experience of self-efficacy; from the participant’s point of view, the prospect of a work group that consists of both patients and staff members and relates to the introduction of RO was motivating and associated with the hope of being able to create something together:

*“Speaker 1: Yes, it’s a pity that Corona was in between, because it’s all relatively far away again. But I remember that it [*meaning the joined training, SSc*] not only motivated people, but also gave them hope; that was a reaction to this event. Hope for a certain degree of self-determination. There was a lot of hope in that. Oh, we can have a say in that, that’s great.*

*Speaker 2: Yes, what also motivated some, uh, um, um, how do you say, is to work in the peer groups and to be able to do that. That was also such a motivational boost: Yes, then I’ll join the peer group”* (FG7, L 25-32).

Nevertheless, the participants discussed whether it is possible to motivate another person to embark his or her “road to recovery”; in general, the participants agreed that the decision to do so might be promoted but cannot be made by another person:

*“He* [referring to the case study used in the training, SSc] *also enforced it. And those who sat next to him in the driver’s seat and said that’s not possible, simply shooed them out once and said, now I’ll take over. That’s what I meant by saying ‘the basic motivation must come from the patient’. Patients must have to want to do it. And then, I think, we can achieve a lot in the interdisciplinary team, even in the setting that we already have”* (FG 3, L 232-235).

Altogether the participants agreed that motivation comes from praise and encouragement and from conveying hope, which is particularly important in case of setbacks, for example, a worsening of symptoms. At the same time, the staff members emphasized that it is very difficult—especially for patients who have committed very serious offenses—to maintain hope for a largely self-determined life.

#### Identity and finding meaning

The elements of “identity” and “finding a meaning” seem to be more difficult for employees to grasp than the other elements:

*“So in terms of connectedness, I can imagine a lot of things you can do. (.) And with the others, I think it is more difficult, because that’s a bit too abstract, isn’t it? About meaningfulness, effectiveness, identity, I think that is perhaps also quite individual*, (…). *Or do you have any ideas about these others?”* (FG 6, L358-369).

Accordingly, the question of how to address these topics was considered to be a challenge:

*“It’s very difficult to find an approach to even deal with this topic, maybe: ‘Who am I? What is my identity?’, isn’t it? I have the feeling that I couldn’t talk about that with many people, even though it is a topic for them. So I’d have to find something, like an indirect route to empower them.”* (FG 6, L. 300 – 303).

Nevertheless, the staff members identified these topics to be important for the patients; however, they might only be addressed individually and in a rather indirect way, whereas the open-mindedness for those topics probably presupposes a certain mental stability on the part of the patients.

Furthermore, the personal identity of the staff members in the therapeutic setting was addressed; in this context, the focus was on discussing and questioning the fact that the staff members usually adapt the function of a role model of moral integrity:

*“Yes, I find that exciting for me. You can always say, there’s the patient, there’s me, I’m free, and he is sick, and they have made a mistake, I’m just the good guy. I am always the good guy. Still, you are also sometimes asking yourself, what are you actually doing with your beautiful evening? What is the meaning of life, do you have it? Are you not interested? You just live, then you go, and goodbye to everyone. So I find that very exciting. Or do I sit alone at home, or do I have a good* [meaning social relationships, SSc] *I thought that is exciting, also for me”* (FG 3, L 500-507).

Moreover, some participants criticized that staff members tend to project their “healthy” standards of norms and values onto the patients, which might not always contribute to the patient’s individual “way of recovery.”

#### Empowerment and participation

From a general point of view, the participants in all focus groups agreed that involving patients in the organization of the living conditions in the wards would result in more self-efficacy and thus promote empowerment:

*“Ah, how can I participate in here? Do I have something to say? And that is also an extremely important experience, in this sense (.) I mean: Ah, okay, we can also change things. We can develop something, we can initiate something. That of course inspires immensely, motivates, when you realize: Aha, okay. There are, yes, you can also, I don’t know, deadlocked things that you can change. You can develop them further. It’s kind of along those lines, isn’t it?”* (FG 6, L 552- 565).

Furthermore, some participants stated that a more active involvement in the wards might motivate the patients to shoulder more responsibility concerning both their lives in the wards and their own therapeutic progress:

*“I have the impression that the more you involve them and resolve this difference, these two sides, the employees, the patients, the more this assumption of responsibility could perhaps also succeed. That one does not speak about them, but with them.” (FG 4, Z. 118-121).*

However, critical voices thereby emphasized that some patients might overestimate their cognitive abilities and underestimate the organizational complexity of the institutional ward conditions, especially regarding the factors “security” and “staff availability.” For instance, some of the patients would be easy to fill with enthusiasm throughout invitations of participation and co-determination but will quickly deteriorate into a state of passivity by recognizing that this also takes the ability to work on problems in a disciplined and concentrated way.

The participants also discussed whether—and if so, to which extent—it is possible to work in a participative manner in a hierarchically structured organization in the first place:

*“We now expect recovery and working groups and to get the patients on board, but in the end I personally consider recovery to be somewhere at the very top of the institutional policy, so I don’t make some decisions, for example, because I know exactly that I am unsure whether I am allowed to take some action steps by my superior or his superior. At the same time, however, I am perhaps now in a working group where I am supposed to encourage the patient to take exactly these steps and this responsibility”* (FG 3, L 340-346).

As this quote demonstrates, there was uncertainty among employees about their own scope of action. The opinion that the patients will become insecure if they have more freedom of decision might be a reflection of this uncertainty of the staff:

*“With some of the patients you can’t, yes, they don’t even want to, because they’ve been in institutions for years, where others decide ‘now you can take a shower’ or ‘now you can get your milk’, or no, ‘now you can do your laundry’, uh, I think (.) we take everything away from them, I always try to take their perspective, how would it be for me, at some point you give up on yourself as a human being, when every day, 24 h, someone decides about you. And then. come and say, now do a project, they don’t dare. Because they also have the fear. at some point someone will say no, many will think, ‘ok now I can always get my razor, or my roll-on deodorant, I no longer have to ask, may I do that’, so yes, I think that will lead to a lot of misunderstandings here and will increase the insecurities of the patients even more.”* (FG 3, L 372-381).

This quote describes basic mechanisms that might result in hospitalism, which—in turn—causes the desire to protect patients from insecurity by patronizing them even in everyday matters.

#### Trust

Most importantly, the staff members declared to be aware that the patients tend to keep potentially important information regarding their state of mind privately due to fear of negative consequences:

*“Patients have also been afraid (.) to say anything at all, out of fear that it could be used against them, or there are also such things, if now someone says, man, I have, I’m, I don’t know quite, I’m suicidal, so yes, then it would mean, earlier, you always went there, if he is suicidal, then he must actually be isolated, if I imagine this, then I think twice, do I say that now.”* (FG 2, L 178 – 184).

In order to counteract this, the participant’s idea was to pay attention to commitment in a more active way. To that end, a climate of clarity in communication must be maintained, for example, concerning the “*a priori*” information, which of the patients’ disclosures must be textually documented and discussed with the team in order not to make the patients feel betrayed if negative consequences arise.

Trust needs to be developed in order to establish an authentic relation, which requires—as a central element of treatment—undergoing commonly shared positive experiences:

*“And I always say to the patients, I try to meet them as I would like to meet them, on an even level, also with boundaries, certainly. And I always say ‘relationship, relationship, relationship’. That is also very, very important for me. And only if I have a sustainable, good relationship something can grow out of it. However, if the relationship is characterized by fear, because of some external influences, it becomes difficult. But we know that.”*(FG 8, L 314- 320).

However, the participants were aware that their relationship with the patients will always be characterized by an institutional power imbalance, which evokes conformity rather than trust and makes the establishment of a trustworthy relation difficult:

*“It is also a different situation in forensic psychiatry than in general psychiatry. This is because there are those with a key and those without. There is a power imbalance, which is difficult to balance*… *or where you have to make extra efforts to counteract that, you can’t just do that. And what else, what I find important in this context, hope goes hand in hand with trust, doesn’t it?”* (FG 4, L 122 – 127).

Establishing a good relationship with the patients despite this power imbalance, which is intrinsic to forensic psychiatry, is cumbersome. However, a variety of more specific obstacles that are not necessarily embedded in the forensic setting make this task even more difficult.

#### Expected obstacles

In all focus groups, the obstacles regarding RO in a forensic setting were a thematically striking topic. These obstacles included social, structural, and personal obstacles.

#### Stigmatization

First, the staff members saw the patients being stigmatized by the juridical and organizational authorities, whose security-centered strict guidelines would have a negative therapeutic effect on the patient’s development, which causes anger and frustration:

*“I think hindrances might be that we are in forensics, we are dependent on the justice system, i.e. on the authorities. That might also be barriers in the work with patients, that one imagines ‘maybe ok, it could now progress a little faster’, but it fails again, and then both must be kept in good humor, so to say, yes, or that the motivation does not fall, so that one can continue to maintain this.”* (FG 2, L 636 – 641).

Moreover—and probably moderated by the stigmatization the patients experience throughout the authorities—staff members also reported self-stigmatization of the patients. The participants expressed their attempts to counteract this noxious phenomenon and expressed their hope of being able to continue doing so with explicit reference to RO:

*“It is often discussed that these mentally ill people often feel excluded from society, and those in our forensic centers even more. And on the one hand, there are already huge inhibitions to the mentally ill from healthy society. But on the other hand, I have the feeling that maybe they don’t dare to integrate. And then I thought to myself, if one could also bring that a little closer, so that they now also have a sense of achievement, in the sense of: ‘Yes, I dared to rejoin this healthy society and nothing happens. It turns out good”’* (FG 6, L 217 – 223).

However, the patients might also feel stigmatized by some staff members:

*“Many patients actually feel hopeless, and I realize that they have questions, and we should accompany them on a path to reintegration and resocialization, but we can’t do that if I don’t know if a patient can really be discharged. How can I give him hope then? It is not possible.”* (FG 8, L 190 - 194).

This quotation demonstrates that some staff members perceive the state of the hopelessness of the patients to be in accordance with their real-life circumstances and tend to refrain from the task of providing hope for a more autonomous future.

#### Structural obstacles

In the psychiatry in Basel, the participants reported obstacles that were related to the general conditions of the premises and the unpredictability of work processes due to unexpected external crisis interventions; due to different constructional and organizational circumstances, these topics were not reported in the focus groups in the other intervention clinic. Moreover (and in both clinics), a shortage of staff was reported to be a problem.

In both adult forensic wards in Basel, staff members experienced the spatial conditions as an inimical stress factor:

*“Speaker 1: That, of course, is a difficulty, it is a high demand, on the patient, logically, but also on us.*

*Speaker 2: So the demands are made, but nothing is done regarding the premises, I think*,… *one tries to improve quality there, quality there, and everywhere is always tried to improve quality, to optimize, but spatially, I think it should be adapted to the current situation”* (FG 1, L 364 - 369).

This obstacle was not very dominant in the forensic ward for adolescents: Although the premises largely correspond to those of the wards for adults in Basel, the occupancy of this ward is significantly lower, which provides more room for the patients. In the focus groups, some participants stated that they can only try to reduce the stress factor of having not enough room throughout compassionate communication, which would, however, not solve the fundamental problem. Building a trustful relation with the patients presupposes the capability to mediate the feeling of being personally welcome, which is not possible if there is hardly any possibility of providing personal space. This shortage of space also leads to messiness in the patients’ rooms:

*“How can I change that, when there are three of me in a small room, that I still have privacy, that I have these small places for retreat that everyone needs, that’s why everything is piled up, because the space itself is minimal”* (FG 3, 530 -532).

Another regularly mentioned obstacle in Basel was the admission of individuals from the penal system who are committed to crisis intervention. These patients cause a lot of restlessness in the wards, as they are unknown to the staff members and require a lot of time and attention, which cannot be contributed to the regular patients; this issue would, for instance, often cause the canceling of meetings with the primary nurse:

*“Yes, when you arrange something with your patient and you realize when you come to work that you’re suddenly in charge of two Isolation rooms, and then you can no longer do certain procedures, which you have planned with him. There must be a lot of understanding from the patient to. yes, aha, I see it, he is in charge of another patient in crisis, now I am secondary, I can’t have much priority, that counters a bit the idea of recovery so to speak, yes”* (FG 1, L 328- 333).

Furthermore, canceling dates due to crisis interventions would include planned leisure activities with the patients outside the clinic, which are held in high regard, as they interrupt the monotony of everyday life and induce joy and motivation.

In contrast to problems that come within a limited space and increased external crisis interventions, the shortage of personnel was problematized in all focus groups.

”*We have not enough offers. So we can’t do all that. That’s not possible. I have a patient now who goes jogging with me for an hour and a half in the sports park. So the relationship is better, the relationship is much stronger. He talks much more, more openly. He is also happy that he can run in the forest. (.) And this experience is missing for many other patients. (.) But we have no staff. So we don’t have the infrastructure to do that”* (FG 7, L 305 -310).

This quote points out that common activities result in a more trusting relationship between staff and patients, which is central to an RO-oriented philosophy of work. However, this is time-consuming and can thus be offered only sometimes to individual patients since there is not enough staff for more complex activities. This lack of human resources caused the fear that introducing RO in Swiss forensic wards might quickly become a “*pro forma*” project, in case agreements between staff and patients cannot be maintained, and patients thus continuously experience frustration. Against that background, the patients might not understand the project as a serious offer and refuse to participate in the corresponding RO work groups.

#### Personal obstacles

Personal obstacles are related to the perspective of the staff members on RO and the patients but also barriers in the minds of the staff members, conflicts among themselves, and missing requirements of opportunities for reflection.

In order to grasp the meaning of recovery, the participants brought forward examples of situations that they considered as being recovery-oriented. On the one hand, a significant amount of participants claimed that RO has implicitly always been practiced in Swiss forensic settings and understood RO simply in terms of rehabilitation and resocialization:

*“Because in principle, every patient who passes through a measure enforcement system because of his will and our support, and can lead a crime-free life afterwards, is actually a recovery story”* (FG 4, L 269-271).

On the other hand, however, a lot of participants self-critically proclaimed the existence of a large, yet unused leeway for conveying the patients to experience more self-efficacy and encouraging them to stand up for their interests.

*“That they dare, so to speak, to think outside the box, or even if they have the liberalization packet, the liberalization order, as we put it, that they think about it, do we like it the way it is, and what is not good, why do we have to make an application, wait three weeks, and then some result comes afterwards, and we don’t really know why.”* (FG 2, L 83).

The belief that the introduction of RO might make a change in that respect is vividly expressed in the following quote. The corresponding participant experiences a deficiency in that same area among patients and hopes that RO provides a basis for addressing this issue:

*“It’s all positive stuff. To me then that is in the sense, because in the everyday life, energy-lessness, hopelessness, it is actually just these things where I experience more in the everyday life, which come much sooner. And that there, of course, you try to give some nourishment, to give a ground, that this -lessness [*emphasis by SSc*] gets away, right? That it goes in the other direction. And here, of course, one may create with all media. Whether that is pastoral care, whether that is everyday life, something, just”* (FG 6, L 68-73).

In the range between these two extreme positions—namely that RO is just another name for the usual work in the forensic wards and that RO can solve central problems of therapeutic relevance that are flagrant yet—the participants expressed several concerns. Some staff members, for instance, were afraid that RO might imply an inversion of hierarchies with the staff at the bottom and the patients on top as the ultimate goal:

*“So now again, what is in my mind after the information meeting, the patient who now has the feeling, ah, this is great, now we can do everything we want and they have to do everything we want, so now if anyone comes up with something, it has to have meaning and purpose, they have to take responsibility, it’s not just we do it so that it becomes more beautiful or better, but it also has to have meaning and purpose, they also have to take responsibility for something they don’t like to do, that’s what I think.”* (FG 1, L 512- 518).

Attempts to negotiate a common understanding of RO occurred in all focus groups. Nevertheless, it was striking that the participants tended to consider RO-related obstacles rather than factors that are unrelated to their own sphere of influence.

First, a major obstacle—from the staff’s perspective—was the patient’s lack of motivation for therapy. In this context, for instance, patients would belittle behavioral problems in their past or even current situations for understandable reasons. This would make it difficult to come to a common understanding as an entry point for recovery-oriented work:

”*I think, what’s such a trouble spot at our place, that is, you can talk about a lot of patients, a lack of insight from the offense, disease, and that makes it very challenging. Partly also very difficult.”* (FG 8, L 71 - 74).

In addition, unawareness of a gap between self-perception and perception by others, which is present in some patients, was also perceived as an RO-starting point “in absence.”

*“That is often [*meaning that patients consider themselves healthier, *SSc] so. I also thought before, I have the feeling that the difficulty is often the completely different perception of everything, right? You don’t have a common ground where you can think about what will help you and what is good for you, right? And the further that diverges, the more difficult it becomes”* (FG 6, L 268- 271).

However, against that background, it is implicitly obvious that the patients “must” adapt to the perspective of the staff from the staff’s point of view. Moreover, no attempts to negotiate different positions in hindsight to the patients’ state of mind were reported or demanded, which was—upon request—attributed to the fact that routines determine everyday life in the wards and there is little time for exchange.

As the following quote suggests, obstacles can also exist in the form of barriers in the minds of the staff members. Such barriers are, for one thing, one’s own values and, for another, the experience that patients might relapse and come back:

*“So one’s own ideas, values, expectations, horizons, experiences with disease patterns, experiences with people with mental illnesses. Unfortunately, we often don’t have the positive examples in the clinic, just those who return again and again, those who commit a crime again, those who stay here for a long time, they are present with us, the others not so much. And from this point of view, we also tend to argue in this way”* (FG 2, L 705- 709).

Changing these own values and ideas is a process that might take a long time, as it implies the necessity of turning away from a paternalistic attitude toward a more open attitude of support, which is directed at detecting what the patients need in order to achieve a goal. Moreover, this process is not only about changing the attitude of individuals because critical elements of the mindset of forensic psychiatry are located on the level of commonly shared staff attitudes:

*“Yes, and I ask myself, what kind of change we all need in our brains, in our attitudes, and how can we achieve it? Can we manage it at all as a whole? It’s not just us, but forensics is also a community, a discipline that also thinks in a direction where we are influenced by education and all kinds of things, like from the outside by the authorities and everything. What do we need, so that we can achieve such a change for ourselves”* (FG 5, L 267 - 272).

Not all of the staff members appreciated this perspective on a possible change; in order to avoid these, it is important to involve all the staff members from the beginning:

*“Speaker 1: But I think that this question [*meaning the question of the necessary change in minds, SSc*] is a very essential one. If we can’t answer that, the whole thing will hit the wall, or it won’t get on its feet as quickly as it could. And that has a lot to do with philosophies of life, with views of life, where do I stand, how do I see the others, how do I see my environment, and maybe you have to put a lot of work into it before you can implement anything in practice on the ward. That you really know everyone behind you and not, now comes the, to make it practical: the early shift is there, and there are, in quotation marks, the ‘right ones’, then comes the late shift. These are the ones who are not yet convinced (collective laughter).*

*Speaker 2: And then it’s all over in the night shift.*

*Speaker 3: In the night shift they close the doors and so, so very practical. If you step on the gas too much, then it’s a complete failure and then the voices that weren’t in favor of it from the beginning get the upper hand again. Yes, that didn’t work, that’s what I said, that it wouldn’t work, right? If we haven’t prepared ourselves well enough in advance”* (FG 5, L 280 - 297).

As this passage demonstrates, there is not always a consensual culture in the specific wards but—in some cases—the presence of different subgroups that pursue their own principles of work: In the corresponding ward, these subgroups can be assigned to the ones that are RO-“convinced,” the skeptics and the opponents. Implicitly, this passage expresses the concern that the project of implementing RO might fail if not most of the staff members do not find a common attitude. For some of the staff, the process of creating and negotiating a common conceptual ground inside the teams is tiring, so they surrender and stick to the simplest solution:

*“Speaker 1: Somewhere you also have to find a middle way, and also come to a common denominator, and ultimately the simplest means is to lock up, lock away, and so on, yes, that’s how the rules come about, from the ward rules and the department rules.*

*Speaker 2: But we have already opened up to a certain extent, right?*

*Speaker 3: But you can’t be sure that it’s going to hold for a month, because depending on, if then somebody doesn’t make it, then suddenly there’s another box of Caotina in the box in the kitchen, so that’s a coming and going with these rules, isn’t it? So the little stories.*

*Speaker 4: I’m sure the patients feel that even more, that’s the one who’s on duty today, with him I know I’ll get what I want and two hours later the other one is on duty and then it’s called.*

*Speaker 1: Exactly that, and then I rather say nothing”* (FG 3, L 452 - 464).

One way to deal with this potential for conflict is to keep questioning ones working style self-critically. However, this is sometimes harder than it seems since it is much easier to locate errors in “the system” instead of questioning oneself. In order to counter such tendencies, a participant brought up the idea of short-term meetings as a room for critique and new ideas:

*“I am firmly convinced that if this basic attitude is not communicated at all, but only a few guards are there, I would say, always in a shift, who then give input again and again. We need reflection aids then, 5 minute meetings, in which one points to such moments completely fast and briefly”* (FG 5, L 310 - 313).

Thereby, the participant already describes a possible solution to how the problem regarding possible conflicts can be met.

### Possible recovery-oriented interventions

The staff members reflected on the possibilities of introducing RO interventions in the wards animatedly and emphasized their motivation to test and implement new ways of thinking off the beaten tracks throughout the project. However, these possibilities presuppose a learning process directed at adapting to the developmental needs of the patients in a more sensitive way and at taking the expertise of the patients into account:

*“So yes, but I think the patients are also to some extent, they know themselves well, and are also professionals for their thing, and I think there is also a bit of a focus, where we have difficulties to say, well, so he knows himself now a lifetime. It is clear that he loses sight of some things now, where it is a matter of offenses, but what is otherwise, if he actually has a psychosis”* (FG 2,L.461-465).

The central themes that emerged discursively together with the patients are related to “orientation to the resources of patients,” “participation,” “ward rules,” “involvement of peer staff,” “creating a therapeutic milieu,” and “shaping of transitions at discharge.”

### Resource orientation

There was consent among the staff in all focus groups that RO requires more than solely a focus on symptoms and tort hypotheses:

*“And I think that we look very, very much at the sick side, the patient is sick and not so much the healthy parts*, strengths, *etc. So that’s what I’m hoping from Recovery, that people will say yes, he does have a lot of healthy parts. We should not only look at the illness, but promote his resources, I think”* (FG 7, L 378 - 382).

Joint activities might help patients to recognize or rediscover their own abilities; congruently, it might allow the staff members to discover the patients from a different perspective. In interacting with patients in a recovery-oriented way, the aim is not to ignore deficits but to encourage patients in their abilities: This mode of working is not a matter of course, and some employees noted that reports tend to be deficit-oriented, for example, in shift handovers, which is attributed to the deficit-orientated performance accounting:

*“Speaker 1: We are often so deficit-oriented in our reporting, and so.*

*Speaker 2: We have to be, that is the guidance system and the diagnostic system for our work, we have to manage this big gap between resource-oriented work, but deficit-oriented documentation”* (FG 2, L 417 - 420).

### Participation

The question of how to involve patients more actively was debated in all focus groups. In this context, one focus group discussed the idea of documenting the progress of the patients together with them. This might increase the challenge of working in a resource-oriented way while documenting in a deficit-oriented manner; however, it envisions the way in which a more participative form of working might work, namely, by involving the patients more actively in their treatment, for example, in site assessments or treatment plan conferences, shift handovers, and ward rounds. In some of the wards, corresponding attempts have already been made, for example, to involve patients in site assessments from the very beginning. The experiences, which have been made in these contexts, demonstrated that this represents a change for all individuals involved:

*“Speaker 1: So we already have a similar method in the Reflecting Team. We actually report with the patient about the patient, and he reports, and everyone reports. But not on a daily base. It takes place once a week.*

*Speaker 2: That’s actually a similar system. We had concerns about that in the beginning as well. Can you speak freely at all? Can you say the same things you would say if the patient wasn’t there? But it works”* (FG 7, L 431 - 437).

For the staff, one of the main difficulties appears to be the question of how to word sensitive issues in a way that makes the patients feel valued and stimulates the motivation to take more self-responsibility, even in the face of critical comments.

### Ward rules

The purpose and complexity of specific ward rules were a topic in all focus groups. The discussion concerned the definition and application of rules from a general perspective and whether the joint revision of these rules would not be a good entry point for recovery-oriented measures. Thereby, the necessary mixture of strictness and flexibility to provide clear orientation but also space for individual interests was discussed. Different positions were examined and considered; in one focus group, a participant presented the following idea:

*“(.) And yes, there are rules, aren’t there? It is quite naive to believe that they do not come, but we simply say that there are rules that have a red dot at the front or back, and there are rules that have an orange or green dot at the front and back, right? That means nothing else than: Green - we can discuss that at any time, orange can be discussed, we have to see how that is, and red, that just stays like that. And this also means, okay, it’s a bit of a*… *there’s a part that can be discussed and debated (.); and there is another part that can’t.”* (FG 6, L 576- 584).

Explicitly, rules for the staff members were also a subject of debate. These referred in particular to security requirements and were considered to be useful. However, the existence of rules with an unknown origin, a doubtful binding force, and a questionable purpose has also been named:

*“Speaker 1: Yes, or activities in general, I mean what we really don’t dare to do is to go out later in the evening.*

*Speaker 2: Yes, but it’s clear that we’re not allowed to do that, so I even know the rules, when it’s dark you’re not allowed to go out in the group [*meaning a group exit in the area, *SSc] anymore.*

*Speaker 3: Yes, but that’s also such an old stuff where*…

*Speaker 2: Yeah, because you can’t see him anymore when he’s running away.*

*Speaker 1: That’s not written down anywhere.*

*Speaker 3: It’s like driving a car, you have to sit in the back seat, you have to sit behind the driver, because the patient can’t reach into the steering wheel, that’s just so clear, been drilled in, or, he’s not allowed in the passenger seat, no way. So now, of course, no more either”* (FG1, L 491-502).

The sense of ward rules thus seems to be questionable in some cases and provides opportunities for a revising recovery-oriented intervention.

### Success stories and stimulation

As a further possibility for an intervention, the staff members anticipated inviting former patients whose life story is a success in terms of recovery. Their assumption was that this might have a far greater effect in terms of promoting hope as if these stories were told by staff members. From a theoretical point of view, these suggestions are in line with the use of peer workers, who work in their fields exactly because they are expected to maintain a more authentic relation with the patients:

*“I met the gentleman [*a former patient, SSc*] the other day and he was really blossoming and really well and beaming. And then I talked to him a little bit, and I asked him what has been his secret. He listed a few things then. I said: Oh man, would you tell that to the adolescents, would you tell that to them? And he said sure, I will, if you support me a little. And I think that’s a great thing, too, and to do that much more. Because it’s something completely different whether we old people say something or whether someone who has just been out for two or three years says something*”(FG 4, L. 510-517).

Furthermore, the participants stated that employees from other institutions might be invited in order to talk about their work and the services they offer in other institutions so that the patients have the possibility to educate themselves about alternative ways of doing things in forensic wards. In addition, it would be a benefit for the staff members if they were in a more vivid professional exchange with other institutions. This might also guarantee a better flow of information, for instance, when a patient is working from the ward at a sheltered workplace.

### Creating a therapeutic milieu

Some staff members assume that there is still a lot of potential room for recovery-oriented work, especially in the ways of interacting with patients throughout primary nursing, where individual interventions might make a big difference since they have a more personal impact due to a very direct relation to the patients:

*“They already have the goal, but they don’t know how to get there, and they need a common thread, just like we do. They simply need to be praised, encouraged on an emotional level, and so on”* (FG 2, L 336-339).

Some already existing group offerings also provide the opportunity to be developed in a direction that captures the idea of RO in a more radical way by giving patients the opportunity and confidence to participate to a higher degree (e.g., in cooking groups) or allowing patients to overtake the role of a leader in other groups.

### Shaping transitions

This topic might have also been presented under the last heading above; however, since it was prominently discussed in two focus groups, it is listed here as a separate topic.

In one focus group, the responsibility to shape a transition regarding the discharge of patients was a controversial subject of debate. Some of the staff members apparently consider this to be solely a task of the social service, while the social worker—who was present in this focus group—would like to see the nurses in a more active role.

In the other focus group, however, the questions of how such transitions should be shaped and how the patients are best prepared for a discharge were raised precisely by the nurses. Thereby, they pointed out that such transitions are shaped in an individual way for each patient, since there are no guidelines for doing so, and there might be some potential for a higher degree of RO in this hindsight:

*“Speaker 1: We always have patients who are in the process of rehabilitation, it’s about the question, are they employable in a field of work*, (…*); the big question always is: How are you prepared for what you have to reveal about yourself outside, if you look at the workplace for example, and you would have to say:’ “I was 5 years in a psychiatric hospital, even longer”*, (…*), and I see that we talk about that in here almost never.* (…*). Can I effectively prepare someone to go into a protected workplace, what does he tell them, what does he tell a colleague, when you get asked, where are you, where do you live, and quite normal questions like this, I think they are actually almost never a topic here, right?* (FG 2, L 136-149).

Regardless of the question concerning the conceptual overlap between RO and rehabilitation, this quotation demonstrates that the need for proper preparation regarding the discharge and an accompanied transition is not always conceptually taken into account in the wards.

## Discussion

Working in a forensic psychiatric ward, an “*often hostile and unpredictable environment*” (29) is inevitably entangled with the challenge of finding a functional balance regarding the tension between security and therapy (13, 14, 30–33). Thereby, situational factors settled within the institutional and juridical framework of forensic psychiatry have a critical influence on the staff’s mode, attitudes, core values, and philosophy of working.

Durcan (34) claimed that “the recovery journey of people in forensic services is significantly different from others,” which is—to some extent—due to the fact that introducing RO as a guiding principle of work in secure forensic wards comes within juridical and technical problems, which are settled in the special features of the field. Johansson and Holmes (35) offered a critical analysis of recovery in secure institutions from a Foucauldian perspective. They conclude that forensic patients must submit to the prevailing worldview of neoliberal personal responsibility. This political power, they argue, is represented by forensic psychiatry, so that patients, despite recovery orientation, have no choice in choosing an individual and personal path to recovery. One may disagree about the understanding of recovery expressed here, but of course, the power structure in a psychiatric forensic institution is not significantly altered by the introduction of recovery orientation.

**FIGURE 1:**
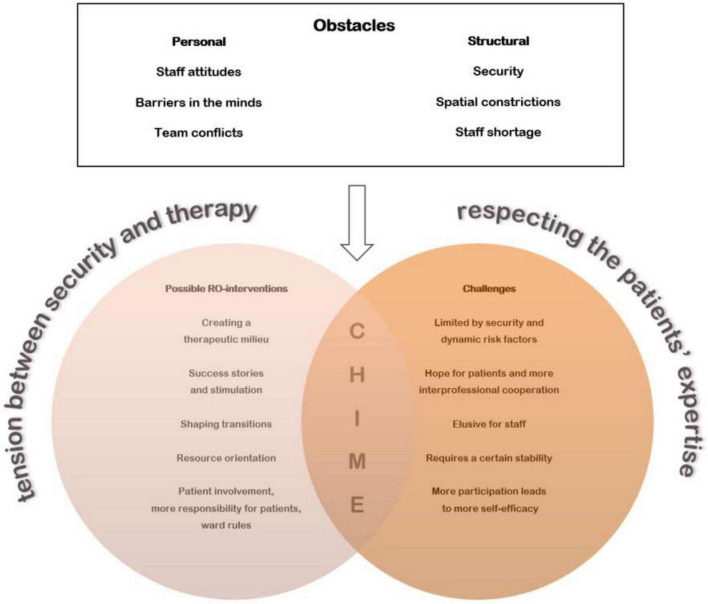
Tentative RE-FOR-MA model of thematic interrelations on secure recovery.

This problem might be insoluble on the level of a single forensic psychiatric institution. However, our analysis provides hints that RO fits the forensic setting on a conceptual level: Thematically, the topics and problems discussed by the staff members did match the five recovery processes, which were developed in general psychiatric contexts. Thus, RO seems to be a model, which is feasible for forensic psychiatry through interventions that relate to the CHIME framework. The conceptual appropriateness becomes evident by the relatedness of the subthemes described earlier to specific elements of the CHIME framework. These subthemes form the thematic overlap between the global themes “expected challenges” and “possible RO interventions.” Thereby, the obstacles influence both the challenges and the possible RO interventions. Figure 1 depicts the themes and subthemes of this study in their relation to the CHIME framework and the expected obstacles (namely structural and personal obstacles) with regard to the introduction of RO in the forensic wards:

In the following, the thematic overlap between the possible RO interventions, the expected challenges, and the specified obstacles is contextualized with the outcomes of already existing literature in that field. According to Tomlin et al. (15), the climate in forensic wards is characterized either by a caring or patronizing work philosophy. Correspondingly, Ewers and Ikin (36) described a role conflict in nursing care in the sense of difficulty for forensic staff members to balance the demands of maintaining a secure environment and the delivery of therapeutic interventions “that often encourage individuals to pursue self-actualization.” Thus, specific recommendations concerning the question of how to come from a rather patronizing to a more caring style of work are discussed subsequently. Thereby, the demand to consider the tension between securing and therapy by respecting the patient’s individual expertise will be highlighted as the central guiding principle.

The CHIME element, “connectedness,” was thematically represented in the anticipated challenge to integrate the patients into socially stable and emotionally valuable relations. The corresponding RO interventions, namely being related to others in a more active way, were a constitutive means for reaching the goal of a higher degree of participation. Furthermore, it offers possibilities for co-production, which can be acquired and developed in a therapy-promoting milieu, or a target state in a supportively shaped transition. The presence of the topic “connectedness” thereby falls in line with the importance of the topic in other studies relating to forensic settings: In addition to being a general key factor in promoting recovery in forensic psychiatry (10), meaningful social relationships have been described as a protective factor concerning criminal recidivism (37–39). Thereby, having meaningful relationships can refer to stable friendships and partnerships, or involvement in various social activities. However, even during a stay in a medium secure forensic ward, the access to family and friends or the possibility to pursue other social activities is fundamentally restricted (40). This makes other patients and staff members on the wards to be the only social surrounding that many patients have (8). Consequently, Mezey et al. (8) argued that this missing opportunity of having social benchmarks outside the clinic makes positive relationships with staff and other patients particularly important. Theoretically, this assumption is in line with Simpson and Penney (5), who describe the relations with the staff as being *“catalytic to the recovery process,”* whereas relationships with fellow patients may be restricted (12, 41). However, the demand for connectedness in a forensic setting might also be detrimentally affected by conditions that are not substantiated in security requirements; in Basel, crowding through spatial restrictions and the high level of noise were perceived as a social stressor. In this regard, it has been demonstrated that a higher occupancy rate and a higher level of noise constitute a stress factor not only for the patients but also for the staff (42), especially if there is not enough room for privacy (11). Thus, the design of forensic hospitals is a central factor regarding both the therapeutic relationship (33) and the job satisfaction of staff members as well as RO on a conceptual level.

How to generate hope was another central subtheme of the anticipated challenges. Hope can be understood in terms of a future orientation with positive expectations that include intentionality, activity, realism, goal setting, and interconnectedness (43, 44). Regarding the interventions, the idea to foster hope by inviting former forensic patients who lead self-determined lives—as possible role models for the patients—was directed at countering the state of hopelessness and resignation that many staff members observe in their patients. Moreover, it became clear that staff members consider “trust” to be a prerequisite for the possibility of generating hope since the quality of the relationship between patients and staff must be on an authentic level that makes patients feel being taken seriously (1, 11, 16, 45–47). Trust can be conceptualized as a process with the characteristic elements of time, reciprocity, and risk and serves to reduce complexity by relinquishing to exercise control over someone else (48). Thereby, transparent communication, a dialogical process, and taking positive risks (49) all contribute to building trust; moreover, specific exceptions from rules against the background of the transparency and the non-arbitrariness of the ward regulations (50) contribute as well. However, in order to make such exceptions, staff members need to know to which extent they are allowed to do so. Especially in forensic psychiatry, this depends on the risk-taking attitude of the institution as a whole, which is a sensitive point concerning the influence of the healthcare system on the adoption of RO (51). Thereby, it also defines the boundaries of the personal risk-taking attitude of the staff members.

As an obstacle that might prevent patients from looking ahead confidently, “stigmatization”—that is, self-stigmatization and the perception of external stigmatization by staff members or other people—was identified as a central problem. The presence of dual (or triple) stigmatization in the experience of forensic inpatients is a well-known fact (8, 10, 12). A study that examined a mediation model regarding internalized stigma in persons with severe mental illness (52) provided evidence that internalized stigma had a negative effect on self-esteem, whereas self-esteem affected hope, and hope affected the general quality of life. Our results suggest that patients might not only feel stigmatized by the legal system or society as a whole but also by the mistrust of staff, or their tendency to consider patients to be too incompetent to live on the basis of self-determination.

Although the CHIME-recovery processes “identity” and “meaning” are described as vital components of recovery (1, 11, 53–55), the staff reported problems with grasping these elements conceptually. Consequently, the participants had no concrete ideas concerning the strengthening of these elements *via* RO interventions. By contrast, protecting and preserving a patient’s identity is described as a central element for mental health nursing in a very common textbook (56) in German-speaking countries, and there is evidence that suggests that nurses tend to pursue these tasks in a rather unconscious manner (57). In the focus groups, the demand for a more active “resource orientation” might be seen as an implicit way to strengthen a patient’s identity since such a mode of working has to focus on competencies and activities that have a positively connoted personal meaning for them.

Not surprisingly, the most concrete idea concerning possible RO interventions related to the CHIME-recovery process is “empowerment.” This notion overlaps with the concept of procedural justice, that is, the idea of fairness in the processes that resolve disputes and allocate resources, which has possible positive effects on criminogenic risk factors (37, 58). The staff members were convinced that more involvement throughout participating in the operational procedures and having a vote concerning the ward rules would result in the experience of self-efficacy in the patients. This claim falls in line with the demand that RO requires a change in the professional relationship from a patronizing structure toward a “partnership working with the patient” (59) as a cooperative or co-productive partnership (60). Self-efficacy is defined as *people’s judgments of their capabilities to organize and execute courses of action required to attain designated types of performances* (61). It can be promoted by positive feedback, experiencing a sense of achievement, learning from role models, and verbal encouragement (62). However, working in such an empowering way with patients requires “enabling spaces for encounter and participation” (63) and even time, which is often missing due to staff shortages. Furthermore, offering the patients more possibilities to participate requires a team culture that has learned to deal with conflicts in a constructive manner regarding the application of rules and the philosophy of work in forensic psychiatry (53) and probably the subordinated support of the management. In this last respect, Le Boutillier et al. [(51), p. 435] found that RO is directly influenced by the *“priorities of the health system, most notably from commissioners and senior managers* (…*)”* and concluded that RO requires commitment from the whole system.

From an integrative perspective, the dominant topic regarding the question of how to establish a more RO caring work philosophy in secure forensic wards was to consider the patients’ expertise regarding themselves under the tension between securing and therapy, as this demand interlinks the paragraphs outlined earlier.

Concerning the development of the required skills for being connected with others or the desired target state to be integrated with a functional social environment, staff and patients must acquire a common understanding of what “being connected with others” or “having a supportive social environment” actually means in case of each individual patient. This, however, presupposes a trustful and hope-inducing relation with the patients, which can be achieved by transparent communication, dialogical processes, and positive risk-taking. Although explicit ideas concerning the induction of meaning in life and identity were missing in the focus groups, RO—which implicitly provides patients with the means to develop their identity and a new meaning in life—substantially implies focusing on the expertise of the patients for themselves.

## Conclusion

The implementation of RO in secure forensic wards in German-speaking Switzerland seems to be feasible. Certain challenges are to be considered, which are specific to the respective targeted unit and institution. These range from structural obstacles in the participating institutions (such as layouts and confined areas and spaces, security procedures, and understaffing) to personal obstacles (such as a fixed mindset, inner team dynamics, and attitudes), as described in the RE-FOR-MA model.

Dealing with these challenges and obstacles is already part of RO and supplements further aspects of the CHIME framework which go beyond the situation in German-speaking Switzerland. Because of its supporting effect on specific forensic treatment goals (which include ameliorating risk profiles, respectively, offender rehabilitation), the effort of implementing RO interventions is worthwhile. For institutions considering RO, part of implementing these involves a further aspect: The general assignment of forensic mental health providers (protection of the public) necessitates security procedures and standards, which have to be balanced with desirable RO interventions, that is, without disregarding potential risks. This issue involves the management of the institution, which is a crucial element in the promotion of RO.

## Strengths and limitations

First, the results of this study correspond to outcomes that have been found in an international context and provide theoretically sound as well as practically feasible approaches for implementing RO interventions in forensic wards.

However, our contents refer to only two forensic psychiatric institutions in German-speaking Switzerland, which—as our results point out—differ regarding central structural characteristics, which are important regarding RO; thus, interviews in other institutions might have revealed other results. Moreover, the participants took part in the interviews of their own free will; therefore, selection bias might have occurred.

## Data availability statement

The raw data supporting the conclusions of this article will be made available by the authors, without undue reservation.

## Ethics statement

The studies involving human participants were reviewed and approved by Ethikkommission der Zentral- und Nordwestschweiz. The patients/participants provided their written informed consent to participate in this study.

## Author contributions

SS: conceptualization, methodology, investigation, writing—original draft, and writing—reviewing and editing. JB: investigating, methodology, and writing—reviewing and editing. AN: conceptualization and writing—reviewing. SR: conceptualization and writing of the review. HH: conceptualization, methodology, and writing—reviewing and editing. All authors contributed to the article and approved the submitted version.
